# The evolving role of cardiovascular magnetic resonance in the assessment of mitral valve prolapse

**DOI:** 10.3389/fcvm.2023.1093060

**Published:** 2023-03-03

**Authors:** Emmanuelle Vermes, Alexandre Altes, Laura Iacuzio, Franck Levy, Yohann Bohbot, Cédric Renard, Francesco Grigioni, Sylvestre Maréchaux, Christophe Tribouilloy

**Affiliations:** ^1^Department of Cardiology, Amiens University Hospital, Amiens, France; ^2^Department of Cardiology, Heart Valve Center, Lille Catholic Hospitals, GCS-Groupement des Hôpitaux de l'Institut Catholique de Lille, Lille Catholic University, Lille, France; ^3^Division of Cardiology, Department of Cardiovascular Diseases, Cliniques Universitaires St. Luc, Pôle de Recherche Cardiovasculaire (CARD), Institut de Recherche Expérimentale et Clinique (IREC), Université catholique de Louvain, Brussels, Belgium; ^4^Department of Cardiology, Centre Cardio-Thoracique de Monaco, Monaco, Monaco; ^5^UR UPJV 7517, Jules Verne University of Picardie, Amiens, France; ^6^Department of Radiology, Amiens University Hospital, Amiens, France; ^7^Division of Cardiology, Department of Medicine and Surgery, Università Campus Bio-Medico di Roma and Fondazione Policlinico Universitario Campus Bio-Medico, Rome, Italy

**Keywords:** mitral valve prolapse, mitral annular disjunction, cardiovascular magnetic resonance, myocardial fibrosis, extracellular volume

## Abstract

Mitral valve prolapse (MVP), characterized by a displacement > 2 mm above the mitral annulus of one or both bileaflets, with or without leaflet thickening, is a common valvular heart disease, with a prevalence of approximately 2% in western countries. Although this population has a generally good overall prognosis, MVP can be associated with mitral regurgitation (MR), left ventricular (LV) remodeling leading to heart failure, ventricular arrhythmia, and, the most devastating complication, sudden cardiac death, especially in myxomatous bileaflet prolapse (Barlow's disease). Among several prognostic factors reported in the literature, LV fibrosis and mitral annular disjunction may act as an arrhythmogenic substrate in this population. Cardiac magnetic resonance (CMR) has emerged as a reliable tool for assessing MVP, MR severity, LV remodeling, and fibrosis. Indeed, CMR is the gold standard imaging modality to assess ventricular volume, function, and wall motion abnormalities; it allows accurate calculation of the regurgitant volume and regurgitant fraction in MR using a combination of LV volumetric measurement and aortic flow quantification, independent of regurgitant jet morphology and valid in cases of multiple valvulopathies. Moreover, CMR is a unique imaging modality that can assess non-invasively focal and diffuse fibrosis using late gadolinium enhancement sequences and, more recently, T1 mapping. This review describes the use of CMR in patients with MVP and its role in identifying patients at high risk of ventricular arrhythmia.

## Introduction

Mitral valve prolapse (MVP), the most common cause of chronic primary mitral regurgitation in developed countries (1, 2), is characterized by the bulging of one or both mitral valve leaflets (with or without thickening) into the left atrium. Its initial prevalence varies from 15 to 35% (3, 4) based on either auscultation or non-specific echocardiography, leading to the overdiagnosis of this pathology. Using the current echocardiographic definition of leaflet displacement beyond the plane of the mitral annulus of at least 2 mm in the parasternal long-axis view (5), the prevalence of MVP has dropped to 2–3% in the general population (6). Although considered to be relatively benign, with excellent survival (7, 8), a number of studies have reported an association between ventricular arrhythmias and sudden cardiac death (SCD), independent of the severity of mitral regurgitation (MR) or ventricular dysfunction (9, 10), with an estimated annual risk of 0.2–1.9% (11–13).

Due to the excellent visualization of the mitral anatomy if affords, its high temporal resolution, and ease of use, two-dimensional echocardiography (associated with transesophageal echocardiography if needed), is the first -line imaging modality for analyzing mitral leaflet motion and thickness and quantifying MR. However, the visualization and quantification of MR can be challenging in cases of poor image quality, especially in the parasternal window, or in the presence of eccentric jets (common in MVP). Moreover, recent studies have suggested a link between MVP, focal fibrosis (in the region of the mitral valve apparatus), and sustained (or complex) ventricular arrhythmia (11) that echocardiography cannot “approach”.

Cardiac magnetic resonance (CMR), with its unique ability to characterize myocardial tissue using late gadolinium sequences, and more recently, T1 mapping, has emerged as a very useful non-invasive imaging modality to assess focal and diffuse myocardial fibrosis, in addition to its accuracy in evaluating ventricular volume and MR quantification (14).

In this review, we will discuss the role of CMR in patients with MVP from diagnosis to LV fibrosis with risk stratification for malignant ventricular arrhythmia and SCD to MR quantification.

### Cardiac magnetic resonance for diagnosing mitral valve prolapse, left ventricular remodeling, and mitral annular disjunction

#### Cine images

Although the spatial resolution of CMR is lower than that of echocardiography, the mitral valve apparatus, including mitral valve leaflets, the mitral annulus, the chordae, and the papillary muscles, can be visualized using balanced steady-state free precession (b SSFP) imaging, ideal for assessing mitral leaflet motion. CMR techniques used to asses MVP are summarized in Table 1. Advantages and limitations of CMR in MVP assessment are presented in Table 2.

**Table 1:** Summary of CMR protocols for MVP assessment.

**CMR techniques**	**Mitral leaflet motion/thickness**	**MA diameter**	**LA measurement**	**MVP measurement**	**MAD/systolic curling motion**	**Focal fibrosis**	**Ventricular volume and function**	**Diffuse fibrosis**	**MR quantification**
SSFP long axis views:									
2-chamber	x	x	x		x				
3-chamber	x	x		x	x				
4- chamber	x	x	x		x				
Short axis (MV level)	x								
Short axis (base to apex ventricles)							x		x
LGE (Dark blood for PM if possible)						x			
T1 mapping/ECV								x	
Phase contrast velocity mapping (sino tubular junction)									x

CMR, cardiac magnetic resonance; MA, mitral annulus; LA, left atrium; MVP, mitral valve prolapse; MAD, mitral annulus disjunction; MR, mitral regurgitation; SSFP, steady state free precession; LV, left ventricle: MV, mitral valve; LGE, late gadolinium enhancement; PM, papillary muscles; ECV, extracellular volume fraction.

**Table 2:** CMR parameters used to evaluate MVP: advantages and limitations.

**CMR acquisition**	**Usefulness/advantages**	**Limitations/pitfalls**
Cine imaging 2-chamber 3-chamber 4-chamber Short axis on the mitral valve	- Assessment of mitral valve apparatus and mitral valve leaflet - Diagnosis of MVP (3-chamber view) and identifying MR - Visualization of MAD - Assessment of mitral leaflet thickness in diastole - Measurement of mitral annulus diameter and atrial dimension - Gold standard modality for ventricular volumes (without geometric assumptions), ejection fraction, LV mass and wall thickness - Highly reproducible and accurate	-Lower spatial resolution than echocardiography (less accurate to assess leaflet thickness and calcifications) - Less sensitive than echocardiography to visualize MR - Time consuming - Less accurate in case of arrhythmic disorders - Limited in patients with breath holding difficulties and claustrophobia
LGE imaging (bright blood and dark blood). Short axis LV stack 2-chamber 3-chamber 4- chamber	- Unique non invasive modality to visualize and quantify focal fibrosis in the myocardium and at the level of the papillary muscles - Predictors of ventricular arrhythmic event	- Requires administration of intravenous contrast agent
T1 mapping. Pre and post contrast (15 to 20'). Short axis view	- Allows measurement of ECV: marker of diffuse interstitial fibrosis	- Requires recent hematocrit value and administration of intravenous contrast agent
2D phase contrast velocity imaging (to measure AFF) and contiguous short axis cine images for LVSV	- MR quantification (MR vol=LVSV-AFF; RF=MR vol/LVSV) - Highly accurate including patients with multiple regurgitation - Independent of jet morphology - Simple equation to calculate regurgitant volume and RF	- Requires two different sequences with their own potential inter-observer and physiologic variabilities - Expertise for selecting the appropriate plane

CMR, cardiac magnetic resonance; MVP, mitral valve prolapse; LV, left ventricle; MAD, mitral annulus disjunction; LGE, late gadolinium enhancement; ECV, extracellular volume fraction; AFF, aortic forward flow; LVSV, left ventricular stroke volume; MR vol, mitral regurgitant volume; RF, regurgitant fraction.

**Figure 1:**
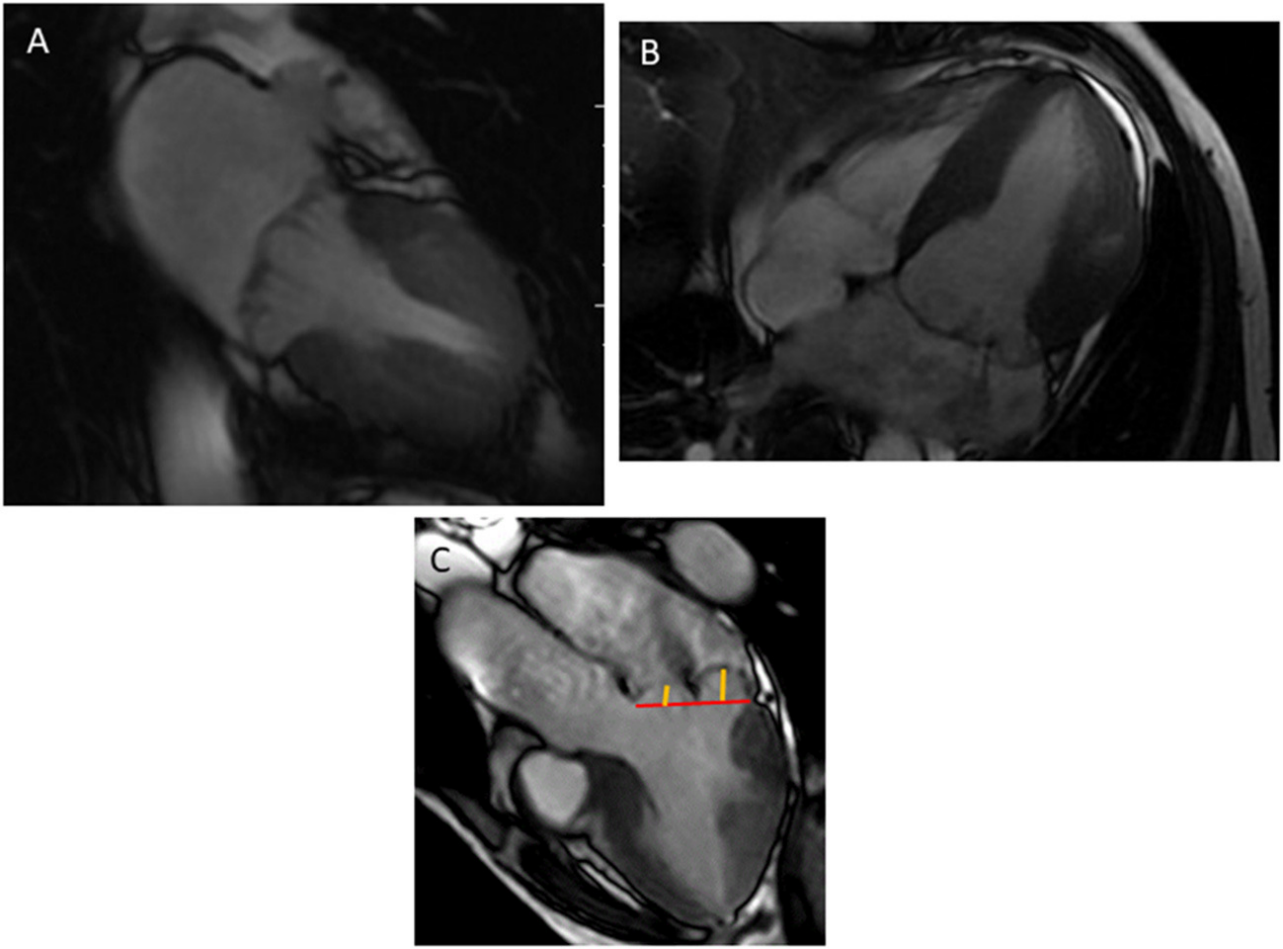
Bileaflet mitral valve prolapse visualized in a 2-chamber **(A)**, 4-chamber **(B)**, and 3-chamber view **(C)**. The annular plane (red line) is positioned, then the measurement of the mitral prolapse is made from the annular plane to the mitral leaflet (orange line).

**Figure 2:**
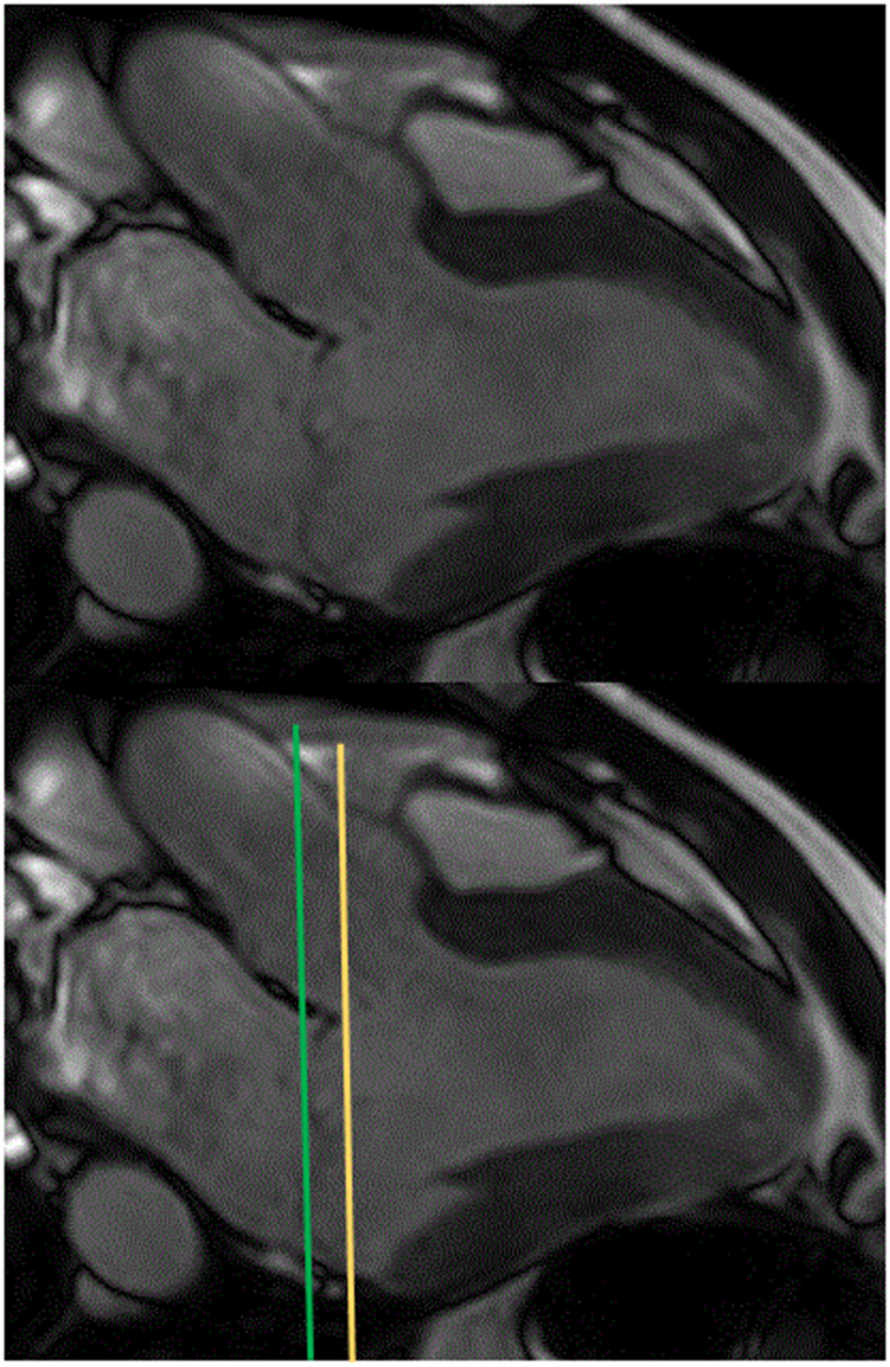
Positions of the left ventricular basal slice at end systole (to trace endocardial borders on short axis images) in 3-chamber view for a patient with a bileaflet mitral valve prolapse: at the mitral valve leaflet level (green line) or at the mitral annular level (recommended) (orange line).

With the use of retrospective cardiac gating (prospective in case of atrial fibrillation), MVP can be visualized in (1) standard long-axis views: 2-chamber, 3-chamber, and 4-chamber; (Figure 1) or (2) a stack of contiguous cine through the plane of the mitral valve, perpendicular to the commissure to identify different scallops of the mitral leaflets.

##### The diagnosis of mitral valve prolapse

MVP is diagnosed using the same criteria of the 2 mm single or bileaflet displacement beyond the long-axis annular plane in a 3-chamber view, equivalent to the trans-thoracic echocardiography parasternal long-axis view (15) (Figure 1).

As in echocardiography, the measurement of leaflet displacement should not be performed in the 2- or 4-chamber view due to the saddle-shaped mitral valve annulus, falsely creating the appearance of a prolapse.

Leaflet thickness is defined as the maximal thickness measured during diastole in long axis views. A thickness ≥ 5 mm defines a classic MVP (or Barlow's disease with myxomatous degeneration of the mitral valve involving both leaflets) vs. non-classic MVP, with a thickness <5 mm (16). However, with a slice thickness between 5 and 7 mm, CMR is less accurate than echocardiography for measuring leaflet thickness.

The mitral annular diameter, commonly greater in MVP (17, 18), is measured at end diastole or end systole in the standard long-axis views (19); its dilatation is often associated with left ventricle (LV) and left atrial (LA) enlargement.

##### Left ventricular remodeling

Due to its ability to measure ventricular volume and mass without the need for geometric assumptions, CMR is the imaging modality of choice for accurately assessing ventricular volumes in patients with MVP. Using b SSFP images acquired during a breath-hold on a stack of short-axis views covering both ventricles from the base through the apex, endocardial and epicardial contours are manually traced at end-diastole and end-systole, including papillary muscles as part of the ventricular volumes. The basal slice at end systole should be at the level of the mitral annulus and not at the mitral valve leaflets, especially for patients with bileaflet mitral valve prolapse (20) (Figure 2).

**Figure 3:**
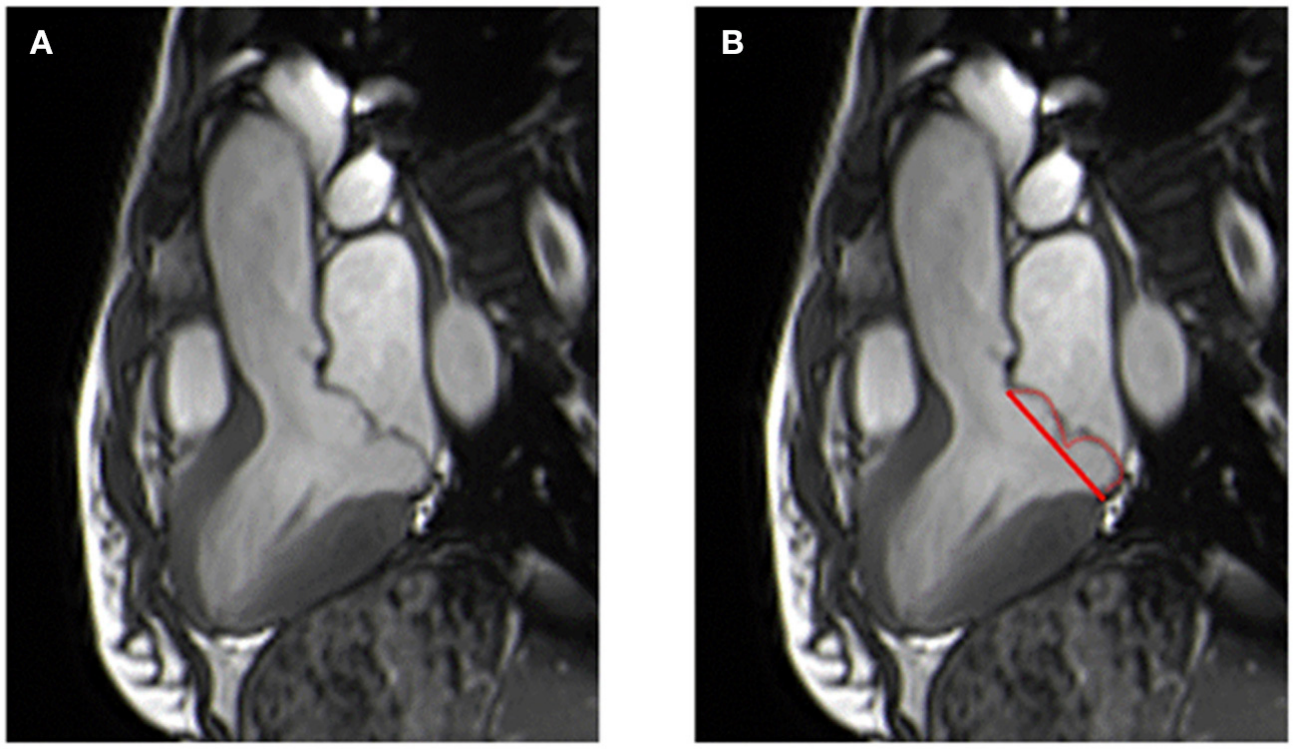
Patient with a bileaflet prolapse diagnosed in 3-chamber view **(A)** with visualization of the prolapse area (red line and curve) **(B)**.

Left atrium contours are traced at end-systole using the biplane area-length method in the 2- and 4-chamber views (21). MVP, especially in Barlow's disease, is associated with higher LV volumes, regardless of the presence of MR (18), leading to the hypothesis that MVP is not only an abnormality of the mitral apparatus but also of cardiac chambers. Levy et al. (22) found that the LV, right ventricle (RV), and LA were significantly enlarged in patients with MVP with moderate or low MR, despite a small mitral regurgitant volume (MRvol) on CMR, with a significantly larger prolapse volume in cases of LV dilatation. The prolapse volume is described as a dome-shaped systolic blood volume between the mitral annulus and the prolapsed leaflets (Figure 3), creating a non-regurgitant volume overload in the LV and the LA that can account for the enlargement out of proportion to the MR in patients with MVP, especially in Barlow's disease (23). El Tallawi et al. (23), measured the prolapse volume of 157 patients with Barlow's disease, in addition to diastolic and systolic volumes, and showed that the indexed LV end-diastolic volume cumulatively increased with increasing MR and prolapse volume. In a multivariate analysis, prolapse volume was an independent and significant predictor of LA and LV dilatation.

LV basal inferolateral hypertrophy has been described and correlates with the degree of annular excursion by CMR (24). Regional LV deformation, assessed by longitudinal and/or circumferential peak systolic strain, has been much more extensively explored by echocardiography than by CMR. Romero Daza et al. (18) were the first to use CMR to demonstrate increased basal circumferential and longitudinal values (especially in the inferolateral and anterolateral segments), regardless of the presence of MR. Abnormal basal inferolateral motion (also called curling) is typically seen in patients with mitral annular disjunction (MAD).

##### Mitral annular disjunction

MAD, is characterized by a separation between the mitral valve annulus-left atrial wall junction and the basal segment of the left ventricular (LV) wall. Disjunction anywhere around the mural leaflet is common and found in 76% of a large cohort of patients without any known cardiovascular disease (25). However, extensive inferolateral disjunction (adjacent to the P2 scallop) is rare (5%). MAD is associated with MVP (25) and independently associated with severe Barlow's disease and LV dilatation (17).

**Figure 4:**
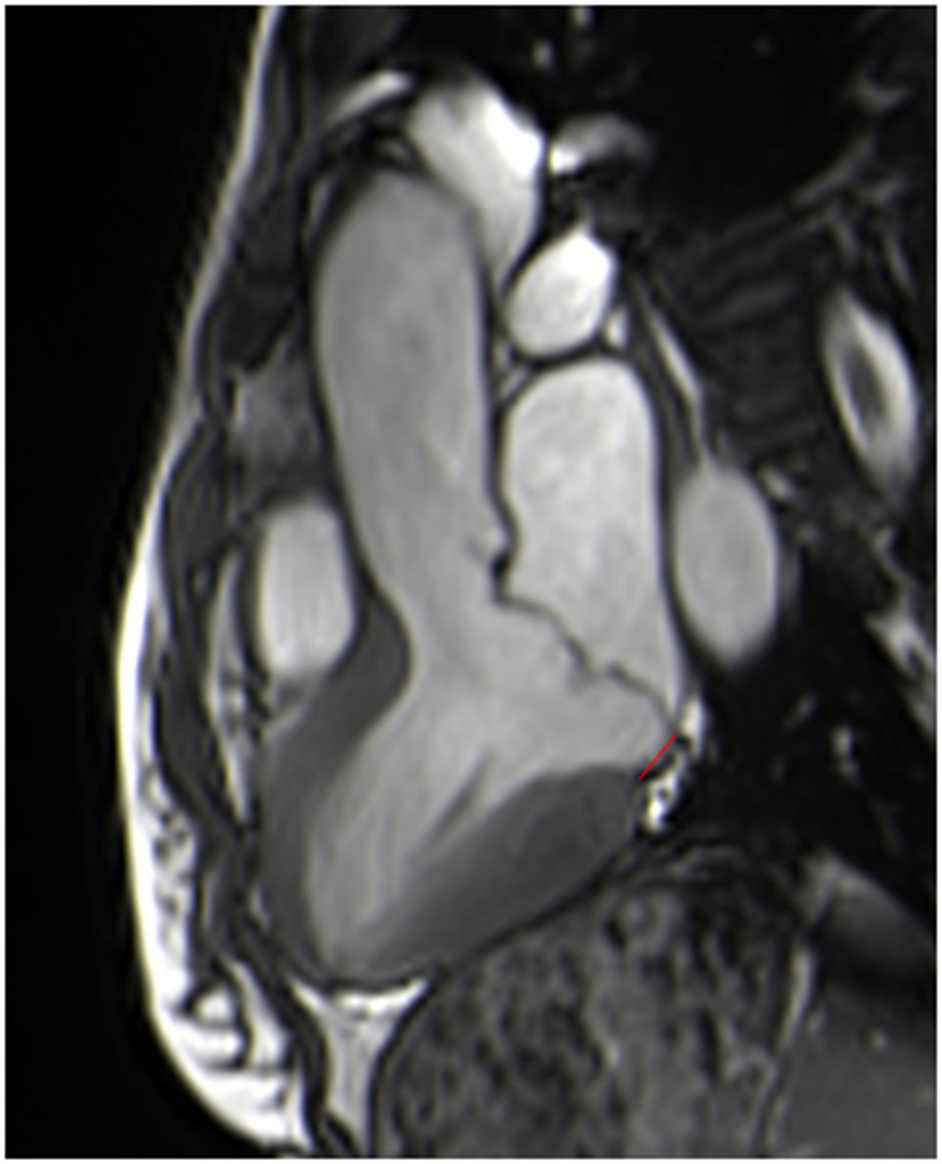
Measurement of the length of the mitral annular disjunction in a 3-chamber view at end systole from the left atrial wall-posterior mitral valve leaflet junction to the top of the left ventricular inferobasal wall (red line).

It can be visualized in end-systole from standard long-axis views as the absence of myocardium between the posterior mitral valve annulus and adjacent basal LV wall. On cine images, patients with MAD show a typical systolic curling motion of the basal LV wall, leading to annular hypermobility (Supplementary Video 1). MAD is present if the maximum distance between the left atrial wall mitral valve leaflet junction and the top of the left ventricular wall is ≥1 mm, regardless of the location (26) (Figure 4). The length of MAD varies from a few millimeters to ≥10 mm.

A recent review that pooled 19 studies found a prevalence of MAD of 32.6% in patients with MVP, increasing to 50.8% for patients with myxomatous mitral valve disease (27). Using CMR as a reference, a recent study (17) reported similar results, with a prevalence of MAD (length measurement 8 ± 4 mm) of 35%, rising to 64% for patients with myxomatous MVP, with a sensitivity of 65% and a specificity of 96% for transthoracic echocardiography. CMR, with its high signal-to-noise ratio, excellent blood-myocardium contrast-to-noise ratio, and reproducibility, may be the imaging method of choice to visualize and measure MAD. Despite its lower temporal resolution, CMR appears to be more sensitive than echocardiography for detecting small MAD <4 mm (28). However, the lack of standardized criteria on how and where to measure MAD using different imaging modalities could result in variability among measurements.

Interestingly, MAD appears to be associated with fibrosis and ventricular arrhythmia (17, 26, 27, 29), especially when the length of the MAD is > 8.5 mm, for the prediction of non-sustained ventricular tachycardia (NSVT), with a sensitivity of 67% and a specificity of 83% (30). MAD has also been reported to be a consistent feature of arrhythmic MVP with LV fibrosis.

### Cardiac magnetic resonance for assessing LV fibrosis

#### Late gadolinium enhancement imaging

CMR, using late gadolinium enhancement (LGE) sequences acquired after contrast administration, allows the assessment of non-invasively regional myocardial replacement fibrosis, a risk marker of arrhythmic events for patients with MVP (31).

Based on a comparison between healthy and fibrotic myocardium, pulse sequences (2D segmented inversion recovery GRE or PSIR or 3D sequences) are performed at least 10 min after gadolinium administration in mid-diastole during a breath hold using the standard long-axis view and a stack of short-axis views (used for LV volume and function) (32). In cases of irregular heartbeat or difficulties in breath holding, single-shot imaging can be performed.

Conventional bright-blood LGE imaging is preceded by a TI SCOUT, set to nullify the normal myocardium. Due to the poor scar-to-blood contrast of these conventional sequences, the detection of subtle papillary muscle fibrosis adjacent to the blood pool can be challenging. By suppressing the blood pool signal, dark-blood LGE imagining could improve the detection of LGE at the papillary muscle level (33). For Dark-blood LGE imaging TI scout is set to nullify the LV blood pool (34).

**Figure 5:**
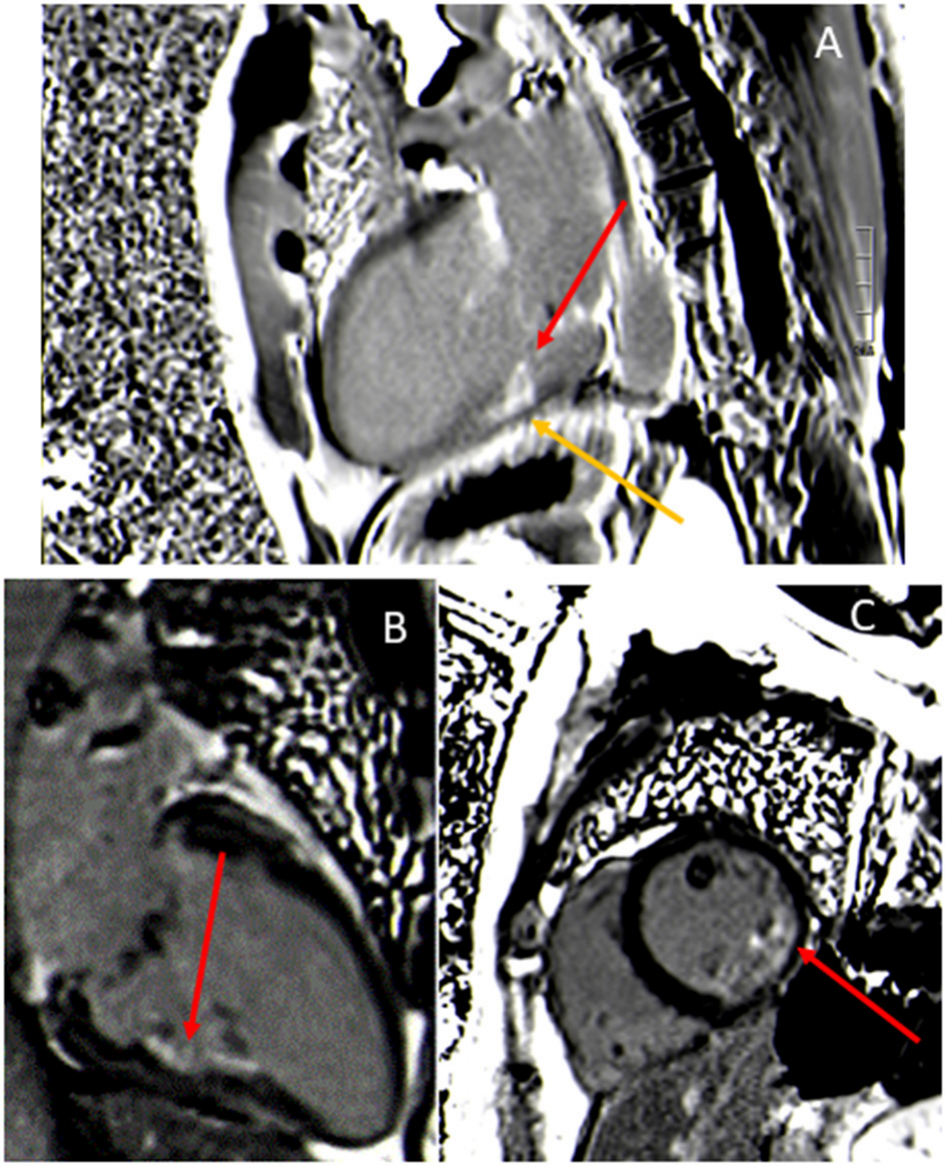
Late gadolinium enhancement images showing enhancement of the posteromedial papillary muscles (red arrows) **(A–C)** and nodular myocardial wall enhancement at the base of the papillary muscles (yellow arrow) **(A)**.

The most common LGE pattern for patients with MVP is a mid-wall or patchy, but non-coronary artery disease-related pattern, mostly visualized in the basal inferolateral or inferior wall and at the level of the papillary muscles (11, 15, 18, 31, 35) (Figure 5). It is well documented that myocardial fibrosis assessed by LGE imaging is a powerful predictor of ventricular arrhythmia in ischemic and non-ischemic cardiomyopathy (36). In MVP, the distribution of LGE in CMR was shown to correlate with histopathological fibrosis (11) and the origin of ventricular ectopy by electrophysiological studies (37). The presence of regional replacement fibrosis can occur early in the course of the disease, found in 13% of patients with only trace-mild mitral regurgitation (MR), increasing to 28% for moderate and 37% for severe MR (35). There is an association between replacement fibrosis on CMR and the presence of ventricular arrhythmia (15, 38, 39). Basso et al. (11) found a prevalence of 93% of LV fibrosis in patients with MVP-related SCD and MVP with complex ventricular arrhythmias. Lee et al. (38) confirmed this association, showing that the volume and proportion of LGE [measured using different methods requiring specific software (40)] were independently associated with sudden cardiac arrest and ventricular tachycardia, as well as MAD. A recent retrospective multicenter study on 474 MVP patients without moderate to severe MR found that only LGE (and not MAD) and preexisting malignant arrhythmias were predictors of an adverse clinical outcome (sustained ventricular tachycardia, SCD, or unexplained syncope) (39).

Excessive mobility of the mitral apparatus (including traction from the chordae tendinae) related to MVP and MAD can induce an abnormal mechanical stress/stretch, leading to the development of hypertrophy and fibrosis in these regions, well documented by histopathology or CMR (11, 41, 42). This mechanically-induced fibrotic tissue may favor the constitution of a re-entry circuit, causing ventricular arrhythmia. The combination of mechanical stretch and myocardial fibrosis of the LV and papillary muscles causes electrical instability, a key role in the genesis of ventricular ectopy (11, 37, 41, 43), reinforcing the role of CMR in its ability to detect MAD, systolic curling, and focal and diffuse fibrosis for predicting patients at high risk of ventricular arrhythmias (44, 45).

#### T1 mapping and extracellular volume

In addition to the detection of fibrotic myocardium, CMR by T1 mapping sequences allows quantification of the myocardial extracellular volume (ECV), a quantitative marker of diffuse myocardial fibrosis that correlates with the magnitude of histological fibrosis (46, 47).

**Figure 6:**
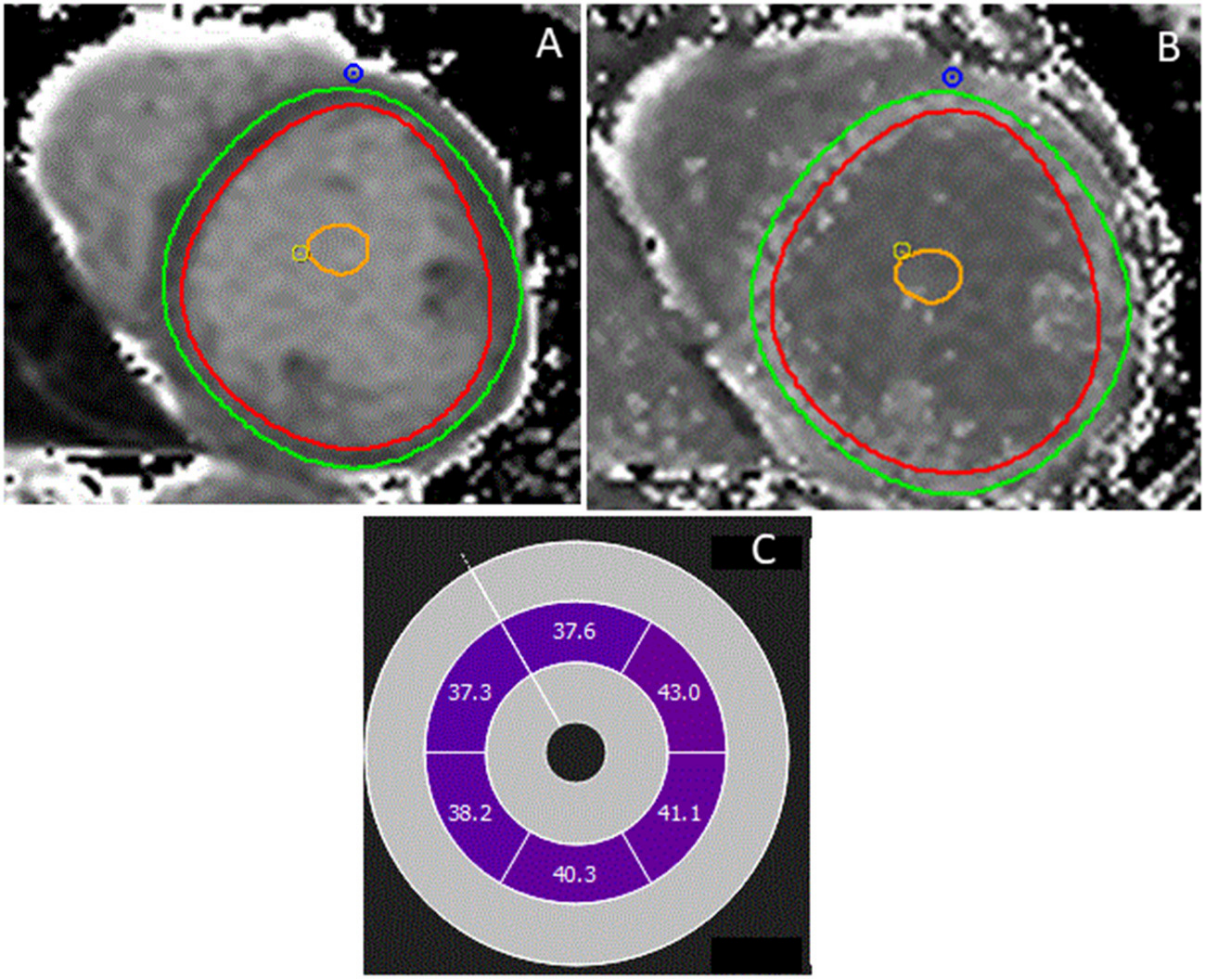
Patient with a bileaflet mitral valve prolapse, mitral annular disjunction, and fibrosis in the papillary muscles. T1 mapping was performed at mid-level before **(A)** and after contrast administration **(B)**, allowing calculation of the extracellular volume (ECV) **(C)**. The ECV was globally elevated (40%) in all segments of the myocardium.

Modified look-locker inversion recovery (MOLLI), shortened MOLLI, or equivalent pulse sequences with motion correction are performed in a single breath hold at the level of the mid-basal and mid-LV short axis before and 15 to 20 min after contrast administration (32). Calculation of the ECV is derived from changes in the myocardial and blood T1 signal (before and after contrast) corrected for the hematocrit, which represents the cellular fraction of the blood (Figure 6). The ECV is less dependent on the magnetic field strength than the T1 signal. In the absence of a hematocrit sample, Treibel et al. (48) have proposed to estimate a synthetic hematocrit based on the relationship between the hematocrit and the longitudinal relaxation rate of the blood.

The ECV has an incremental diagnostic benefit in non-ischemic cardiomyopathy and correlates strongly with outcome, better than native T1 mapping (49).

In MVP, a recent retrospective study by Pavon et al. (50) that used the synthetic ECV (ECV syn) showed it to be higher in patients with MVP and MAD than in controls (87% had a mean ECV above the upper limit of 27%). However, focal fibrosis increases the ECV and could represent a potential confounding factor. Interestingly, the authors showed that 81% of LGE negative MVP-MAD patients had an ECV syn >27%, suggesting that fibrosis is present in the myocardium of all patients and not only in those in which it is detected by inferobasal LGE. Moreover, they showed that among patients with complex ventricular arrhythmias, the ECV was increased for all patients with MAD but only in 53% of those with LGE suggesting that interstitial fibrosis (and not only focal fibrosis) could be a predictor of ventricular arrhythmia in MVP-MAD patients (50). Indeed, electrophysiological studies have shown that ventricular arrhythmias in MVP originate not only from papillary muscles but also from the outflow tract or fascicular regions (51).

In a large prospective study of 229 patients with MVP and 195 without, Kitkungvan et al. (52) showed that the ECV (at mid-level) increases with MR severity, regardless of MR etiology (prolapse or not) and was independently associated with MR-related symptoms and MR severity on multivariate analysis. Chronic LV volume overload could also play a role in diffuse interstitial fibrosis development in MR.

### Cardiac magnetic resonance for quantifying mitral regurgitation

#### Cine imaging and cine phase contrast velocity mapping

There are three different CMR methods (two indirect and one direct) to quantify MR severity in MVP, all independent of geometric assumptions, unlike echocardiography.

**Figure 7:**
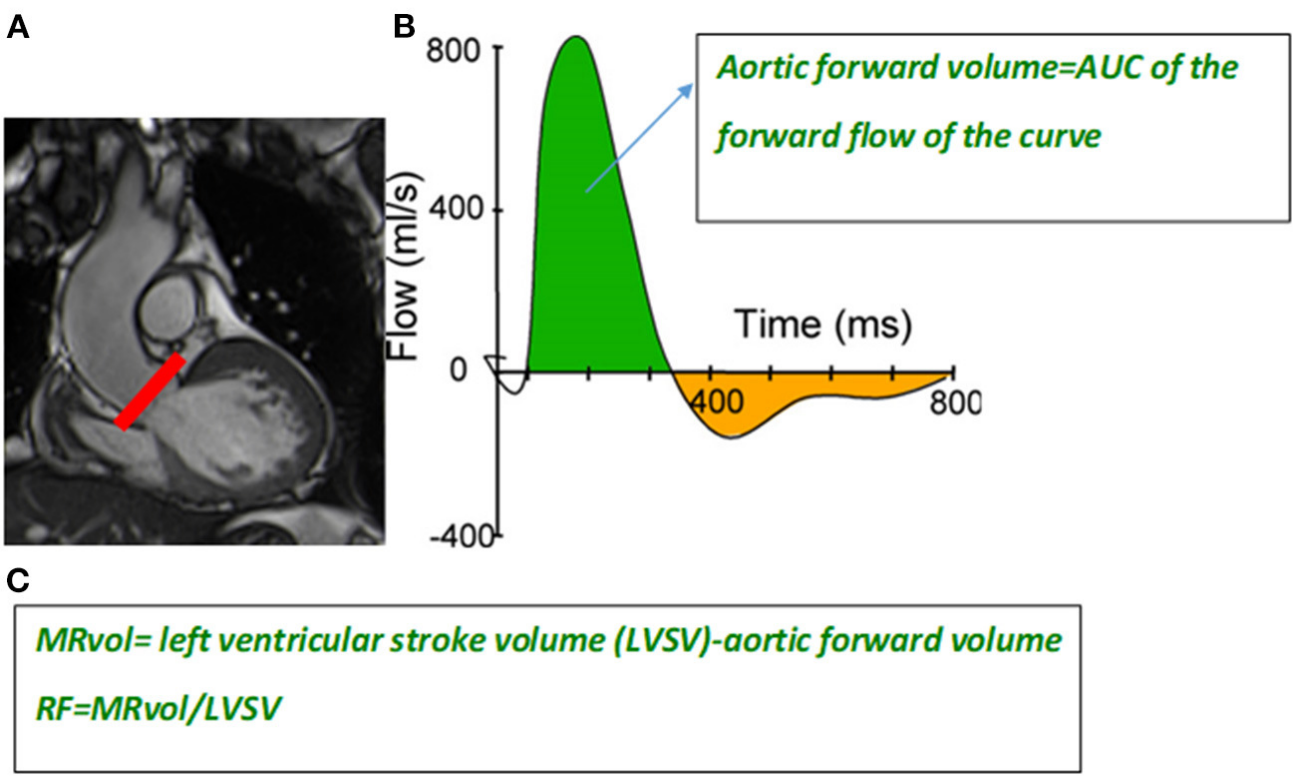
Slice position at the level of the sinotubular junction (red line) **(A)** to perform a phase-contrast velocity mapping sequence to generate a flow curve **(B)**. The aortic forward flow volume is calculated as the area under the curve (AUC) of the forward flow on the flow curve (green area). Formula to calculate the mitral regurgitant volume (MRvol) and regurgitant fraction (RF) **(C)**.

The most recommended method is indirect, requiring measurement of the left ventricular stoke volume (LVSV) and aortic forward flow (AFF) to calculate the MRvol and regurgitant fraction (RF) (Figure 7). The two other methods (14) are less used in clinical practice and will not be discussed further.

The LVSV is expressed as the difference between the LV end diastolic volume (LVEDvol) and LV end systolic vol (LVESvol), calculated using manual contouring in end diastole and end systole, including that of trabeculae and papillary muscles on short-axis cine SSFP images covering the entire LV. The basal slice is defined as that in which the LV myocardium is detected at a minimum of 50% of its circumference, corresponding to the level of the mitral valve annulus. The AFF is obtained from phase-contrast velocity mapping performed in the proximal ascending aorta above the tip of the aortic valve and perpendicular to the vessel. A flow curve is generated allowing calculation of the area under the curve of the forward flow curve (Figure 7).

The MRvol is defined as follows: LVSV-AFF and RF = MRvol/LVSV.

This method, which is highly reproducible and valid in cases of multiple valvular regurgitation, is independent of the shape and morphology of the jets, which is particularly useful for patients with late systolic and eccentric regurgitant jets for which delimiting the radius of the converging hemisphere is difficult in echocardiography (53).

Several studies have shown consistent discrepancies between echocardiography (PISA method) and CMR in MR severity grading (54–56), especially for patients with eccentric jets with a higher MRvol by the PISA method (54, 55). A recent study on 188 patients with MVP assessed by echocardiography (PISA and volumetric method) and CMR, confirmed a weak correlation between PISA-MRvol and volumetric methods either by CMR or echocardiography (0.29 and 0.30, respectively, *p* < 0.001) (54). Applying the echocardiographic CMR cutoff (MRvol ≥ 60 ml for severe MR) would result in 32% (60 patients) of the patients being reclassified from severe (with PISA) to moderate MR with CMR. Interestingly, the overestimation of MRvol with PISA decreases with MR severity and a reverse trend is observed among patients with MAD, higher LVEDvol and large prolapse volume (22, 54).

In patients with bileaflet MVP, positioning of the basal slice in CMR at the mitral annulus (also called the “functional method”) (20) means that the blood trapped between the annulus and leaflets is not included in the LVESvol estimation, leading to a higher LVSV, MRvol, and EF than that estimated using the “anatomical” method (at the mitral valve leaflets). Better correlations between the LVEDvol and MRvol (*r* = 0.79 and *r* = 0.67, respectively) and the LVEF and global myocardial strain (*r* = 0.86, *r* = 0.68 respectively) have been shown using the functional the anatomical method (20). However, in a recent study, the MRvol determined by CMR or echocardiography correlated with the LVEDvol (*r* = 0.68 and *r* = 0.66, respectively) unlike for the RF (54). As MRvol is linked to LV size, the use of an arbitrary cutoff of severity by CMR is likely to be inappropriate, as LV remodeling differs between patients with severe MR. Therefore, the MRvol measured by CMR should be interpreted with caution in clinical practice to avoid underestimation of MR severity in the presence of truly severe MR but with limited LV enlargement, as frequently encountered in older patients and women. As the RF normalizes the MRvol to the size of the LV, the RF over MRvol may be preferable for quantifying MR. Although large trials on specific CMR thresholds are lacking, a recent review of the literature suggests a cut off of RF ≥ 40% for severe MR (14), similar to a recent study using 4D flow CMR (57).

#### 4D flow

Unlike 2D flow, 4D flow is possible in a single continuous free-breathing volume acquisition (7–10 min) covering the entire heart and allowing visualization of flow in multiple orientation with a possibility to retrospectively measure any flow in any direction.

Regurgitant volume and fraction can be derived from direct measurement of regurgitant flow (through plane regurgitant flow calculated from pultiplanar reformatted planes at the peak velocity of MR) or from indirect 4D flow quantification (58) (4F flow mitral forward flow – 4D flow aortic stroke volume).

4D flow imaging has demonstrated promising results with good agreement in secondary MR (59) but low agreement in primary MR with a trend to exaggerate regurgitant volume. Indeed, eccentric jet can be challenging to identify and follow through out systole.

Data on 4D flow in MVP are scare. A recent study on 54 patients with MVP assessed in transthoracic echocardiography, standard volume CMR and 4D flow (direct and indirect methods) showed a good correlation between methods (r = 0.59–0.84, *p* < 0.001) with the best concordance between standard CMR and 4D indirect method (60). Compared to echocardiography, in a cohort of 33 patients with chronic primary MR, Ribeyrolles et al. (57) proposed CMR thresholds of RF ≤ 20% for mild MR; 21–36% for moderate MR and ≥37% for severe MR to get the best agreement between these two methods (Kappa=0.9: 95% CI 0.7–0.9).

#### How to select patients with MVP for CMR?

In all MVP patients, clinical evaluation, personal and family history, ECG and echocardiography should be the first steps before CMR. As CMR is not an imaging modality always available and echocardiography is the first line technique, we would recommend addressing patients for CMR in case of inconclusive or uncertain echocardiography on MVP and MAD assessment or MR severity discrepancies between MR severity on echocardiography and clinical finding. In case of arrhythmic MVP (complex premature ventricular contractions on ECG or holter monitoring, frequent inverted T-waves in the inferior leads or severe myxomatous disease), a systematic CMR is recommended to identify specific arrhythmic risk factors such as focal or diffuse fibrosis.

## Conclusion

CMR has become an important component of MVP assessment due to its accuracy in assessing ventricular dimensions and function, mitral annulus motion, and mitral regurgitation severity, in addition to its unique ability to non-invasively assess focal and interstitial diffuse fibrosis.

With use of cine images in standard long-axis views, MVP can be visualized and quantified, as well as mitral annular disjunction and prolapse volume. Using an indirect method that combines LV measurement and phase-contrast velocity mapping in the ascending aorta, MR can be quantified with a high level of accuracy using the regurgitant fraction and mitral regurgitant volume.

With the use of late gadolinium enhancement sequences and T1 mapping, focal and diffuse fibrosis, with calculation of the extracellular volume, can be detected and quantified, providing useful information to identify MVP patients with a high risk of ventricular arrhythmias.

As a complement to clinical and echocardiographic data, CMR, using a complete protocol as described above, should be integrated into the workup of patients with MVP.

## Author contributions

EV provided design of the research and wrote the manuscript. EV, LI, CR, and SM provided iconography. AA, LI, FL, YB, CR, FG, SM, and CT provided critical revision of the manuscript. All authors contributed to the article and approved the submitted version.

## Supplementary material

Supplementary Video 1 Cine 3-chamber view showing mitral annular disjunction in a patient with bileaflet mitral valve prolapse.
